# Differential Interactome Based Drug Repositioning Unraveled Abacavir, Exemestane, Nortriptyline Hydrochloride, and Tolcapone as Potential Therapeutics for Colorectal Cancers

**DOI:** 10.3389/fbinf.2021.710591

**Published:** 2021-09-14

**Authors:** Hande Beklen, Sema Arslan, Gizem Gulfidan, Beste Turanli, Pemra Ozbek, Betul Karademir Yilmaz, Kazim Yalcin Arga

**Affiliations:** ^1^ Department of Bioengineering, Faculty of Engineering, Marmara University, Istanbul, Turkey; ^2^ Department of Biochemistry, School of Medicine, Marmara University, Istanbul, Turkey; ^3^ Genetic and Metabolic Diseases Research and Investigation Center (GEMHAM), Marmara University, Istanbul, Turkey

**Keywords:** colorectal cancer, drug repositioning, differential interactome, prognostic markers, systems biomarkers

## Abstract

There is a critical requirement for alternative strategies to provide the better treatment in colorectal cancer (CRC). Hence, our goal was to propose novel biomarkers as well as drug candidates for its treatment through differential interactome based drug repositioning. Differentially interacting proteins and their modules were identified, and their prognostic power were estimated through survival analyses. Drug repositioning was carried out for significant target proteins, and candidate drugs were analyzed via in silico molecular docking prior to *in vitro* cell viability assays in CRC cell lines. Six modules (mAPEX1, mCCT7, mHSD17B10, mMYC, mPSMB5, mRAN) were highlighted considering their prognostic performance. Drug repositioning resulted in eight drugs (abacavir, ribociclib, exemestane, voriconazole, nortriptyline hydrochloride, theophylline, bromocriptine mesylate, and tolcapone). Moreover, significant *in vitro* inhibition profiles were obtained in abacavir, nortriptyline hydrochloride, exemestane, tolcapone, and theophylline (positive control). Our findings may provide new and complementary strategies for the treatment of CRC.

## Introduction

Colorectal cancer (CRC) is the most commonly diagnosed cancer in women and men worldwide. It occurs in the colon or rectum and affects the large intestine or large bowel. It is also known as bowel cancer, rectal cancer and colon cancer. The predicted new cases are over 1, 9 million and the number of deaths is 9,35,000 in 2020 (Sung et al., 2021). It is assumed to increase to 2.2 million new cases and 1.1 million deaths by 2030 (Douaiher et al., 2017). According to American Cancer Society, only 4 out of 10 CRC patients are detected at an early stage. If detected at an early stage, the 5-years survival rate can be as high as 90%. Otherwise, if metastases occur in all parts of the body, the 5-years survival rate drops to 14% (Rahib et al., 2014).

For many years, various drugs have been improved for the treatment of CRC, 5-fluorouracil, bevacizumab, cetuximab (Mauricio, 2019) also in combination with 5-fluorouracil/leucovorin with either oxaliplatin (FOLFOX) or irinotecan (FOLFIRI) (Neugut et al., 2019). However, patients with advanced CRC are resistant to 5-fluorouracil (Douillard et al., 2000). Therefore, new therapeutic agents may be needed for treatment.

In the last decades, many systems biology approaches have been used to overcome problems in the diagnosis, prognosis, or therapy of many cancers including CRC (Turanli et al., 2017a). For instance, five potential target genes (DUSP4, ETV5, GNB5, NT5E, and PHLDA1 to overcome cetuximab resistance in CRC utilizing gene expression profiling and mathematical modelling, and the results were subsequently validated through wet-lab studies (Park et al., 2019). Furthermore, several source genes and target proteins were identified through the employment of differential gene expression analysis, protein-protein interactions (PPIs), and genome-scale metabolic modelling (Zhang et al., 2019; Ilyas et al., 2020).

Physical interactions between proteins in all living organisms are the basis for cellular signaling pathways that mediate essential biological processes such as gene expression and metabolism (Sevimoglu and Arga, 2014; Calimlioglu et al., 2015). By integrating high-throughput genomic data with PPI networks under the concept of network science, disease mechanisms and genes associated with diseases or proteins can thus be discovered. Clarification of disease mechanisms associated with biological processes and identification of disease candidate genes or proteins through the use of PPI networks may suggest potential biomarkers and drug targets for these diseases (Safari-Alighiarloo et al., 2014).

Biomarkers play a crucial role in the concept of personalized medicine in identifying subtype phenotypes, identifying the convenient therapeutic approach and estimating clinical course and prognosis. Due to the limited efficiency of molecular biomarkers in the diagnosis and treatment of complex diseases such as cancer, researchers have recently focused on the detection of biological clusters of molecules (mostly gene and/or protein clusters) that have functional relationships with each other and are referred to as systems biomarkers (Turanli et al., 2017a). Accordingly, studies have shown that the identified systems biomarkers play an active role in the diagnosis and prognosis of diseases, as well as in the development of treatment strategies (Gov and Arga, 2017; Kori and Arga, 2018; Sevimoglu et al., 2018).

The differential interactome approach relies on important alterations that appeared in protein-protein interactions (PPIs) between phenotypes. The differential interactome algorithm allows to predict the probability distributions for each possible co-expression profile of gene pairs (encoding proteins that interact with each other) across phenotypes and to determine the uncertainty of whether a PPI matches the corresponding phenotype (Ayyildiz et al., 2017). This approach has shown success by being utilized effectively in various cancers and subtypes. (Ayyildiz et al., 2017; Turanli et al., 2019a; Turanli et al., 2019b; Gulfidan et al., 2020; Caliskan et al., 2021).

Drug repositioning (DR), in which existing drugs are repurposed for a new therapeutic indication is a promising approach because it reduces time and cost in drug development and circumvents problems due to safety and efficacy issues (Shim and Liu, 2014; Würth et al., 2016; Xue et al., 2018). Recently, a review paper pointed out repositioning in drug-resistant CRC (Nowak-Sliwinska et al., 2019). Citalopram, amantadine, and captopril are repurposed drugs for the prevention or treatment of disease (Koh et al., 2014; Van Noort et al., 2014; Diaz-Carballo et al., 2015). Also, there are computerized efforts to reuse drugs such as GW-8510, etacrynic acid, ginkgolide A, and 6-azathymin with the use of Functional Module Connectivity Map for CRC (Chung et al., 2014). Currently, the identification of candidate biological targets and new potential drugs could be done by using *in silico* methods for DR by collecting clinical data at different omics levels and analyzing them in systematic and integrative pipelines (Turanli et al., 2019a). As an instance, the well-established drug metformin is used to treat Type 2 diabetes and was found to be a preventative agent in CRC via in silico methods (Higurashi and Nakajima, 2018). In addition, signature-based DR is another strategy in the DR approach to identify existing drugs for potential treatment or to fulfill new indications. This method considers gene expression signatures and compares drug-gene and disease-gene expression profiles. (Yella et al., 2018). One of the earliest examples of signature-based approaches is “Connectivity Map” and a public resource used to find small molecules and mechanisms of their action, chemicals or physiological processes, diseases, and drugs (Lamb et al., 2006). Another category of DR is network-based DR, which utilize to identify molecular mechanisms and diagnostic/prognostic biomarkers in many diseases including cancer. Besides, network modelling is an important approach for computational drug repositioning, forming a triangle of disease, genes, and drug (Yella et al., 2018).

Considering the urgent need for the development of new therapeutic strategies in CRC, in this study, we applied a network-based DR approach to propose novel drug candidates for CRC treatment. For this purpose, taking into account the fact that colorectal carcinomas arise predominantly from adenomas, we evaluated comprehensive datasets for human colorectal adenomas and carcinomas together, employed the differential interactome algorithm and evaluated the potential of differentially interacting proteins as drug targets. Then, we repurposed candidate drugs which are later analyzed *in silico* by molecular docking simulations and *in vitro* by using viability assays to determine their potential in CRC treatment.

## Methods

### Gene Expression Datasets

Two comprehensive transcriptome datasets were employed: 1) the microarray dataset (GSE8671) (Sabates-Bellver et al., 2007) which compiled on Affymetrix Human Genome U133 Plus 2.0 Array platform (Affymetrix Inc.,Santa Clara, CA, United States) with 32 colorectal adenoma samples and 32 matched normal tissue samples obtained from NCBI-Gene Expression Omnibus (GEO) (Barrett et al., 2013), and 2) the RNA-seq COAD and READ datasets (normalized as FPKM) from The Cancer Genome Atlas (TCGA) consisting of 644 tumor tissue samples, and 51 normal tissue samples (Tomczak et al., 2015). TCGA-COAD consisted of 478 primary tissue samples and 41 normal colon tissue samples. TCGA-READ consisted of 166 primary tissue samples and 10 normal rectal tissue samples.

### Protein-Protein Interactions Data

BioGRID database (v.3.5.167) (Chatr-aryamontri et al., 2017) was used, which contains 35,688 physically and experimentally detected PPIs among 8,570 human proteins for the human protein interactome. Filtering the interactome dataset with proteins encoded by the genes represented in the transcriptome datasets resulted in a network of 34,603 PPIs among 8,322 proteins considering the TCGA dataset and 32,259 PPIs among 7,951 proteins considering the GSE8671 dataset.

### Identification of Differential Interactome and Differentially Interacting Proteins

The differential interactome algorithm was recruited to identify the differential PPIs (dPPIs) between the tumor (or adenoma) phenotype and the normal phenotype, considering the relative observation frequencies (q-value) of each PPI as previously described (Ayyildiz et al., 2017; Gulfidan et al., 2020). Briefly, genes were grouped into three different levels as −1, 0, 1 according to their expression levels within each sample. When the expression level of a gene was lower than the average expression value, it was labeled as “−1’; when the expression level of a gene was higher than the average, it was labeled as “1”; otherwise, it was labeled as “0”. The false discovery rate was 0.05. According to the three-level expression categorization, there were nine possible gene expression states (i.e., [0 0], [0 1], [0 −1], [1 0], [1 1], [1 −1], [−1 0], [−1 1], [−1 −1]) for each interacting protein pair. The number of times the conditions occurred in the normal group (N_0_) and the number of times they occurred in the tumor group (N_1_) were calculated. Taking into account the imbalance between the sample sizes of the groups, the count parameters were normalized considering the total sizes of the normal (N_N_) and tumor (N_T_) groups (the maximum possible numbers of N_0_ and N_1_, respectively), and the normalized observation frequencies in each group were obtained. The q value (the estimate of the probability of that state occurring in the tumor state) was calculated as follows:

$$q=\;\frac{\frac{N_{1}}{N_{T}}}{\frac{N_{0}}{N_{N}}+\frac{N_{1}}{N_{T}}}$$

PPIs with a normalized observation frequency in either the normal or tumor phenotype greater than 20% and q-values of less than 0.10 (significantly repressed in the tumor phenotype) or greater than 0.90 (significantly activated in the tumor phenotype) were considered dPPIs. Differential interacting proteins (DIPs) were defined as proteins that exhibit significant alterations in their interaction patterns during the transition from normal phenotype to tumor state (Gulfidan et al., 2020), and categorized into two groups depending on their interaction patterns: 1) DIPs with repressed interactions under tumor conditions, and 2) DIPs with activated interactions under tumor conditions. Modules were constructed around DIPs using their dPPIs and visualized using Cytoscape 3.7.2 (Shannon et al., 2003).

### Gene Set Over-Representation Analysis

Gene set over-representation analyses were carried out using the ConsensusPathDB (ver.34) (Kamburov et al., 2013). Preferred data sources for metabolic and signalling pathways were KEGG (ver.88.0) (Kanehisa et al., 2017), Reactome (ver.64) (Fabregat et al., 2017), and Biocarta (ver.2009_05_12) (Nishimura, 2001), and Gene Ontology annotations (Ashburner et al., 2000) were employed to represent associated biological processes. Statistical significance was defined by a *p*-value cut-off < 0.05 for all functional enrichment analyses. Each *p*-value was subsequently converted to a z-score by using the inverse normal cumulative distribution.

### Prognostic Power Analysis

The prognostic analyses were performed using two datasets, GSE17536 (Smith et al., 2010) and TCGA-COADREAD containing patient survival data. The dataset of GSE17536 consists of 177 samples (24 patients with stage I, 57 patients with stage II, 57 patients with stage III, and 39 patients with stage IV) obtained from a patient cohort having the average age of 65.5 ± 13.1, whereas the dataset of TCGA COADREAD consists of 466 samples (89 patients with stage I, 170 patients with stage II, 130 patients with stage III, 62 patients with stage IV, and 15 patients with no stage information) acquired from a patient cohort having average age of 66.9 ± 12.5.

Survival analyses were performed by stratifying patients into high- and low-risk groups based on their prognostic index (PI), which is the linear component of the Cox model (PI = β_1_x_1_ + β_2_x_2_ + … + β_p_x_p_, where *β*_p_ is the coefficient obtained from the Cox fit, x_p_ is the expression value of each gene) to investigate the prognostic performance of each DIP module. Analyses were performed with the web server SurvExpress (Aguirre-Gamboa et al., 2013) using datasets with clinical data. Signatures of survival in each risk group were estimated by Kaplan–Meier curves and Hazard Ratios (HR). The statistical significance of each curve was assessed by the cut-off for the log-rank *p*-value < 0.05. The hazard ratio (HR = (O1/E1)/(O2/E2)) was calculated to discover the significance of the survival curves based on the ratio between the relative death rate in group 1 (O_1_/E_1_) and the relative death rate in group 2 (O_2_/E_2_), where O denotes the observed number of deaths, and E denotes the expected number of deaths.

### Drug Repositioning

GeneXpharma (Turanli et al., 2017b), which is a publicly available platform presenting 50,304 gene-drug interactions among 4,344 genes and 11,939 drugs and employing statistical tests for the disease-gene-drug triad, was used for the network-based DR considering protein targets CDKN2A, GSK3B, HDAC2, and PML. A hypergeometric probability test was used to associate drugs with target proteins, and simulation results with *p* < 10^−3^ were accepted as statistically significant.

### Molecular Docking

The 3-D structures of target proteins were obtained from Protein Data Bank (PDB) (Berman et al., 2002). The PDB identifiers were 1DC2 (CDKN2A) (Yuan et al., 2000), 6Y9R (GSK3B) (Buonfiglio et al., 2020), 6WBZ (HDAC2) (Yu et al., 2020), and 5YUF (PML) (Wang et al., 2018). Ligand binding sites of proteins were determined by PDBe (Velankar et al., 2016) and BIOVIA Discovery Studio (ver.20.1) (BIOVIA, 2016). The structures of candidate drugs were taken from PubChem (Kim et al., 2019). Autodock Vina software (ver.1.1.2) (Trott and Olson, 2019) was used for molecular docking analyses. All water molecules were deleted, and all polar hydrogens were added to the structure of a protein in the preparation of macromolecules. Molecular docking was performed five times for each ligand and each simulation yielded twenty poses. Exhaustiveness was set to eight for all docking calculations. Binding affinities were estimated to determine the importance of binding between protein targets and drug candidates. The top-scoring pose (with the lowest binding free energy) was selected for further analysis.

### Drugs

Abacavir (Selleckchem, S5215), Ribociclib (Selleckchem, S7440), Exemestane (Selleckchem, S1196), Voriconazole (Selleckchem, S1442), Bromocriptine mesylate (R&D systems, 0427/50), Tolcapone (Selleckchem, S4021) were dissolved in dimethyl sulfoxide (DMSO) and then diluted in 1x PBS. For each treatment step, 100 mM stock solution was diluted to a final concentration. Total DMSO concentration did not exceed 0.1% and control groups (CTRL) were also treated with the same concentration of DMSO. Nortriptyline hydrochloride (Selleckchem, S3698) and Theophylline (Sigma, T1633) were dissolved in 1x PBS.

### *In Vitro* Cell Viability Assay

CCD-841-Con cells (ATCC, CRL-1790, human healthy colon epithelial cell line) and HCT-116 cells (ATCC-CCL-247, human colorectal carcinoma cell line) were cultured in Dulbecco’s modified Eagle’s medium and Eagle’s Minimum Essential Medium, both supplemented with penicillin (100 units/ml), streptomycin (100 g/ml), and 10% fetal calf serum in a humidified atmosphere of 5% CO_2_ and 95% air at 37°C. Later, cells were treated with varying abacavir, voriconazole, nortriptyline hydrochloride, tolcapone, and theophylline concentrations (10, 50, 100, 150, and 200 µM) in the first phase. Another wide exposure dose was used as 10, 50, 100, and 200 nM and 200–500 µM for bromocriptine mesylate, budesonide, exemestane, voriconazole, and theophylline. Also, nortriptyline hydrochloride concentration in the range of 10–50 µM was used to identify IC_50_. Cell viability was determined using MTT (3-(4,5-dimethylthiazol-2-yl)-2,5-diphenyl-2H-tetrazolium bromide) reduction by viable cells following the exposure durations of 24, 48, and 72 h 2 µl of MTT solution was mixed with 100 μL medium and incubated for 3 h at 37°C and 5% CO_2_. At the end of this incubation period, the medium with MTT was discarded and 100 μL DMSO was added. The formazan crystals were dissolved by shaking at 150 rpm for 10 min. The intensity (OD) of the color formed was measured with a spectrophotometer with a microplate reader at a wavelength of 590 nm (Reference filter; 660 nm). Calculations were performed related to the absorbance of control samples which was equaled to 100%.

## Results

### Differentially Interacting Proteins, Their Modules, and Biological Interpretation

**FIGURE 1:**
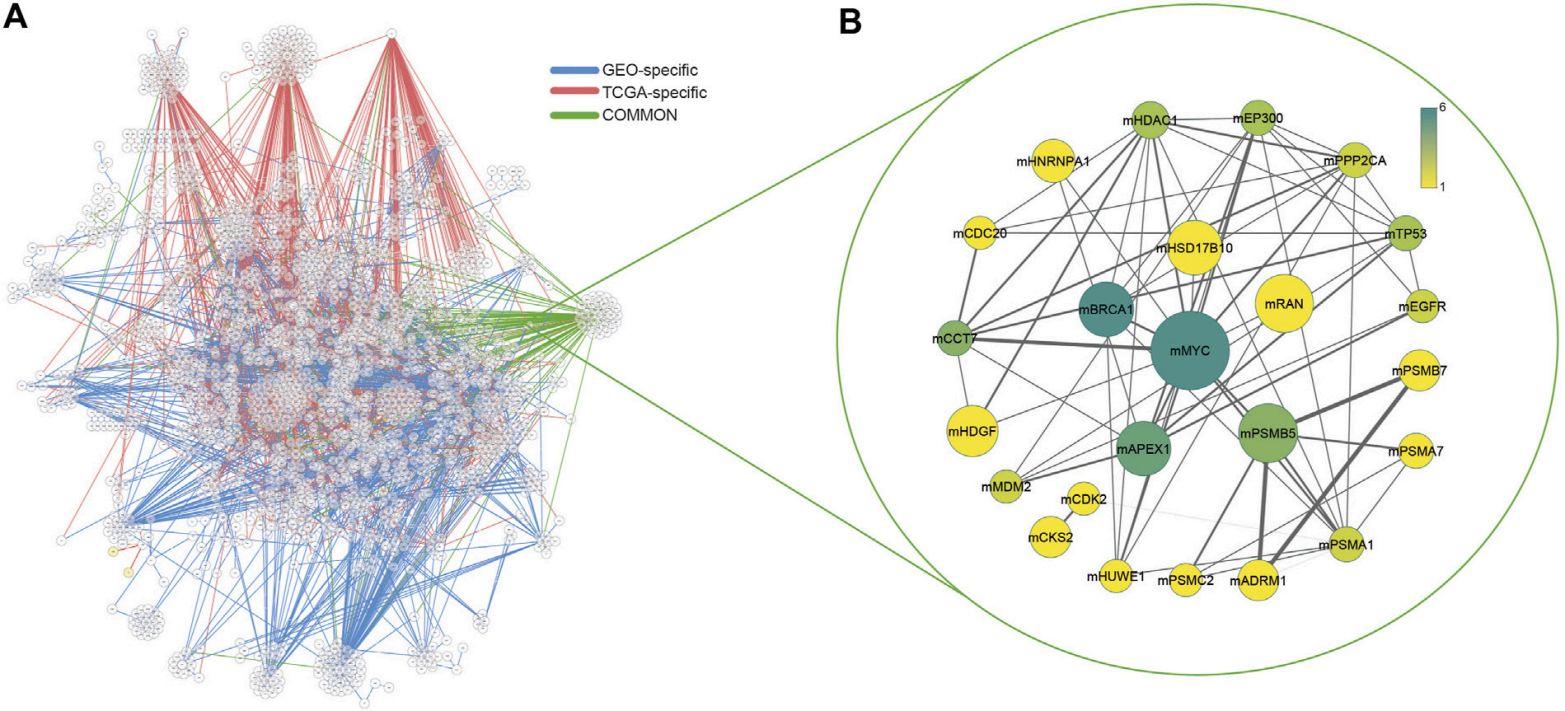
**(A)** Interaction networks of both datasets and common interactions. GEO-specific edges were shown in blue, TCGA-specific edges were shown in red and common interactions were shown in green. **(B)** Common modules in both datasets. Node size increases with the size of each module, edge thickness increases with the number of shared interactions between modules. The color scale of nodes changes according to the presence of each hub protein in other modules.

Two comprehensive transcriptome datasets associated with colorectal adenomas and tumors were recruited to apply differential interactome methodology for predicting high probability PPIs in tumor states and identifying differential PPIs. Two datasets were analyzed independently, and common dPPI signatures were considered in further analyzes. In the current formulation, upper and lower bounds (0.90 and 0.10, respectively) were used for q-values which represent the probability estimates. PPIs that had a normalized frequency of observation in either the normal or tumor phenotype greater than 20% were considered as significant. As a result, a total of 2,214 differential PPIs (dPPIs) were identified in the GEO dataset and 1,625 dPPIs were identified in the TCGA dataset. dPPIs were further classified as “significantly repressed in tumor phenotype” (if *q* < 0.10) or “significantly activated in tumor phenotype” (if *q* > 0.90). To this end, among the dPPIs, 718 interactions were repressed and 1707 interactions were activated in the GEO dataset, while 81 interactions were repressed and 1,557 interactions were activated in the TCGA dataset (Figure 1A).

The scale-free topology of the differential interactome network highlights the presence of hubs termed differentially interacting proteins (DIPs), indicating major changes in their interaction patterns during tumorigenesis (i.e., transition from “normal” to “tumor” phenotypes) (Ayyildiz et al., 2017). In this study, we identified DIPs and constructed a module around each DIP with its interaction partners, and the corresponding module was named adding the letter “m” to the beginning of the central DIPs (e.g., mMYC). The number of modules with more than 5 PPIs was 86 in TCGA and 177 in the GEO dataset. In both datasets, modules with at least 5 PPIs were considered and filtered based on their common interactions only. The number of common modules in two datasets (GEO and TCGA) were 24 (Figure 1B, Supplementary Table S1).

Among these modules, mMYC was the largest module with 116 members. Besides, the MYC protein was the most common protein observed in other modules. Moreover, mPSMB5, mCCT7, mRAN, mAPEX1, mHSD17B10, and mHDGF were also modules with more than 10 interactions. mPSMB5 had 33 interactions, as well as 30 interactions for the mCCT7, and mRAN had 29 interactions. For others, the number of interactions was 15 and 11, respectively.

**FIGURE 2:**
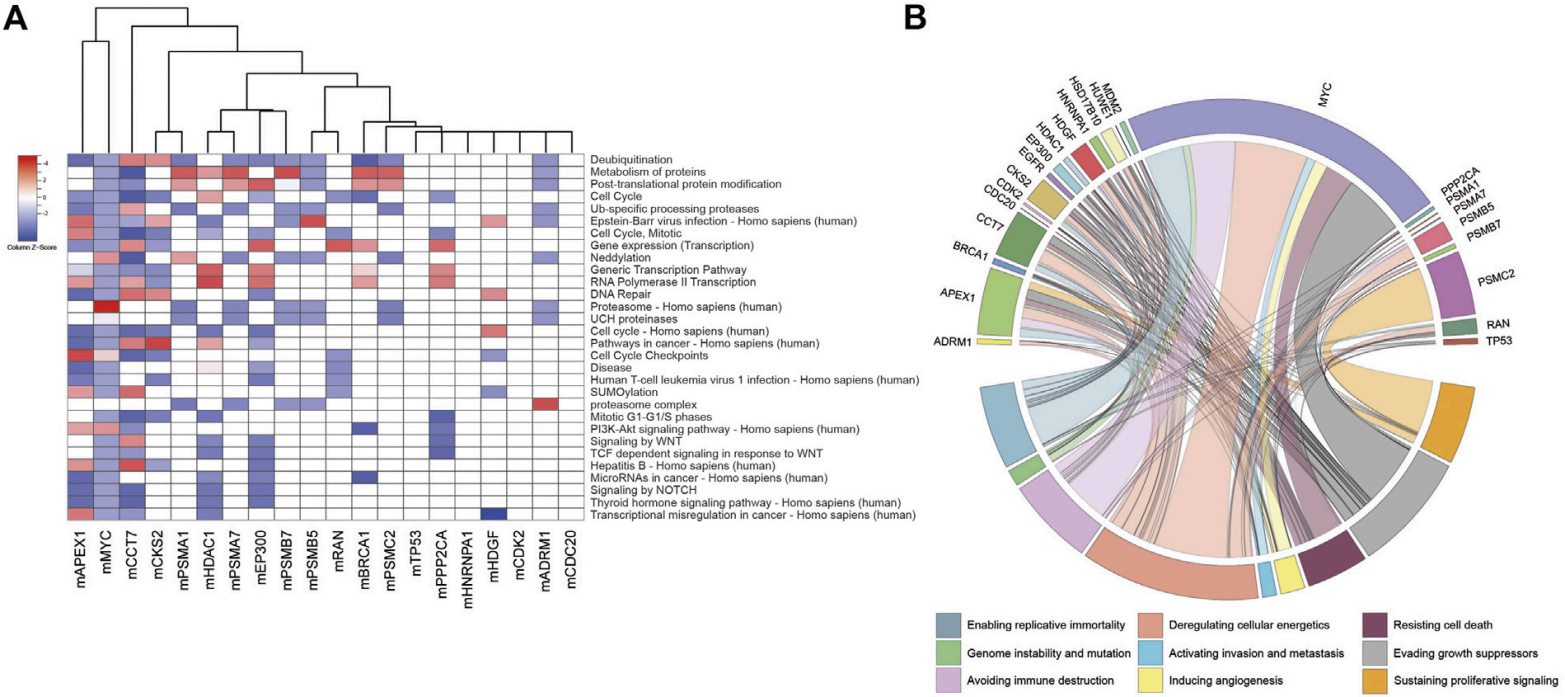
**(A)** Heatmap indicating top-scored 30 pathways enriched with the modules. Cells were colored depending on statistical significance (i.e., z-score). **(B)** Circos plot linking cancer hallmarks to modules via the number of module members associated with the cancer hallmarks.

Considering the members of the modules, gene set over-representation analyses were performed to identify biological processes, molecular pathways, and cancer hallmarks associated with the DIPs (Figure 2, Supplementary Table S1). The members of the modules were significantly enriched with several biological processes such as deubiquitination, protein modifications, cell cycle, neddylation, and transcription; signaling pathways such as PI3K-Akt, NOTCH, and Wnt signaling; cancer pathways such as transcriptional misregulation of cancer and microRNAs in cancer; and cancer pathways associated with oncogenic viruses such as Hepatitis B, Human papillomavirus (HPV), Epstein-Barr virus, and Human T-cell leukemia virus 1 (Figure 2A, Supplementary Tables S2, S3). Furthermore, the members of the modules were significantly associated with all cancer hallmarks (Senga and Grose, 2021), i.e., sustaining proliferative signaling, inducing angiogenesis, resisting cell death, deregulating cellular energetics, evading growth suppressors, activating invasion and metastasis, enabling replicative immortality, avoiding immune destruction, and genome instability and mutation (Figure 2B).

### Evaluation of DIP Centered Modules as Potential Prognostic Systems Biomarkers

**FIGURE 3:**
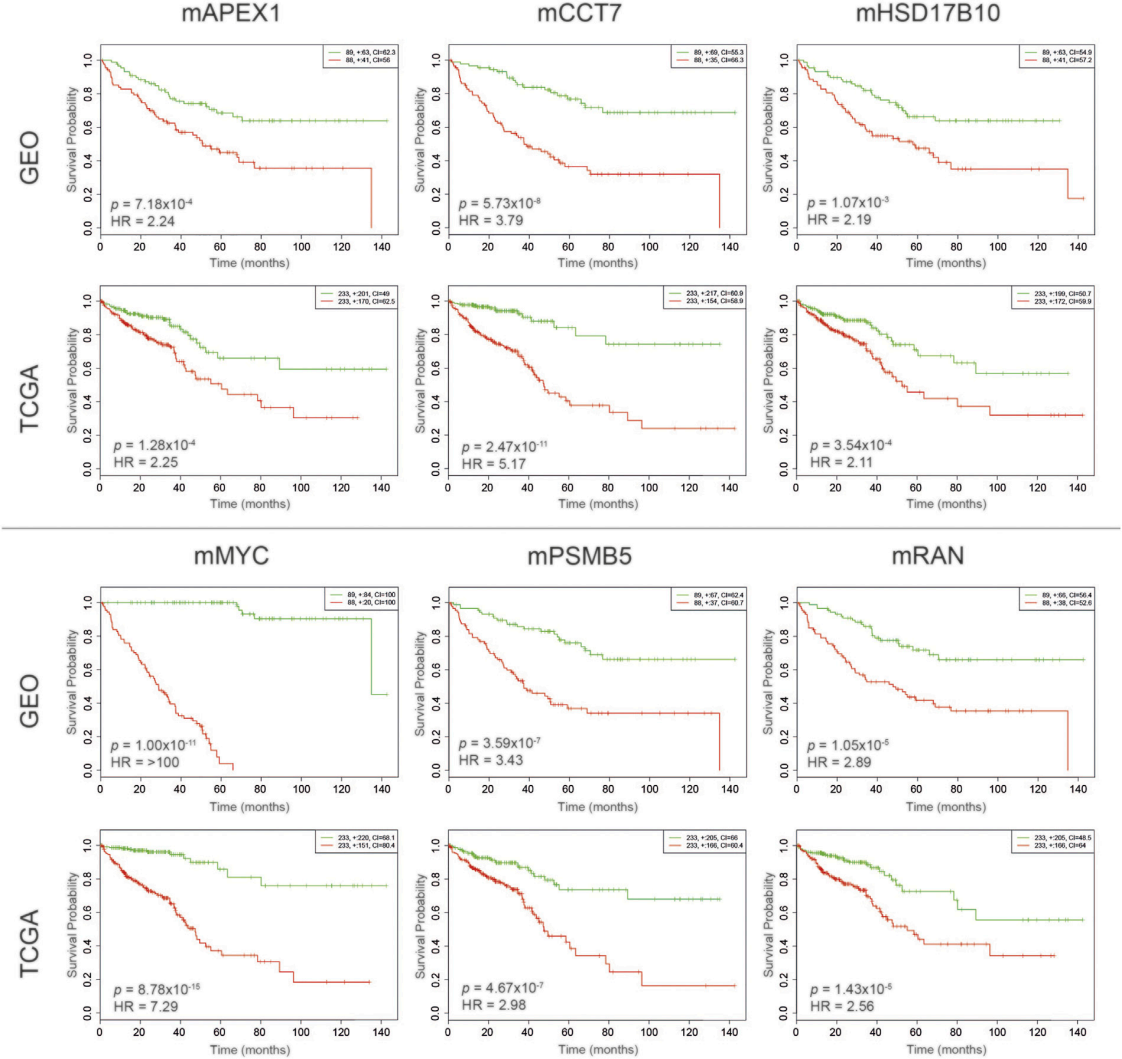
Kaplan–Meier plots estimating survival probabilities of patients considering GEO and TCGA datasets. Patients were stratified considering their prognostic index determined through gene expression profiles. The low-risk group was shown in green, and the high-risk group was shown in red.

The evaluation of prognostic performances of DIP-centered modules was performed via Kaplan-Meier survival analyses through partitioning low- and high-risk groups regarding the expression levels of module genes. To cross-validate the results, the survival analyses were independently evaluated using two different datasets. As a result, prognostic performances of six modules (namely, mAPEX1, mCCT7, mHSD17B10, mMYC, mPSMB5, and mRAN) were found significant in both CRC datasets considering log-rank test p-values (*p* < 0.01) and hazard ratios (HR > 2) (Figure 3).

### Identification of Candidate Drugs for CRC Treatment Through Drug Repositioning

**FIGURE 4:**
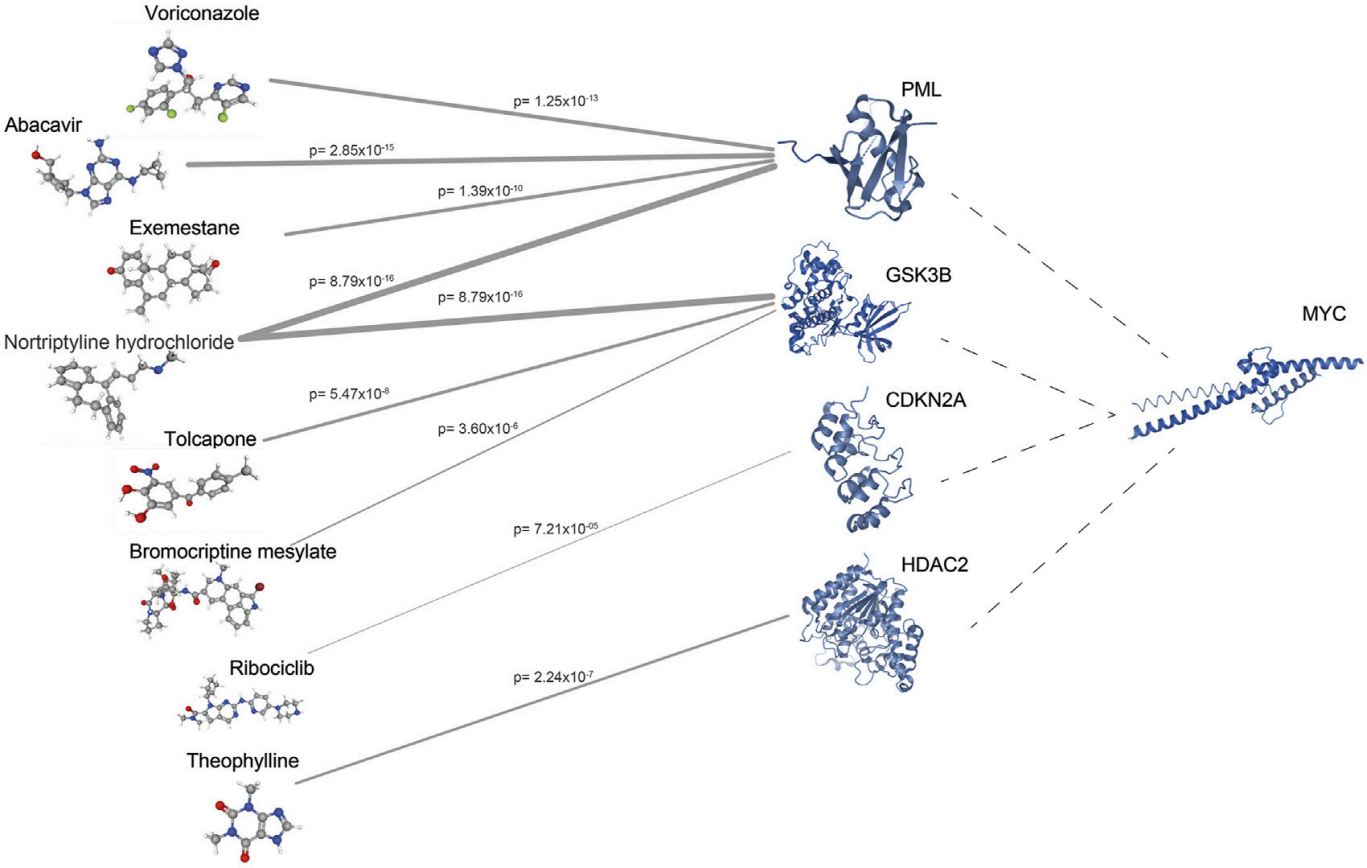
The network between drug targets and drugs obtained from geneXpharma. Edge thickness varies according to the hypergeometric probability (p) describing the significance of the drug-target association.

To identify potential drug targets, modules were further evaluated considering their sizes, members, prognostic performances, and enriched processes, pathways, and cancer hallmarks. Considering its relevance, we focused on the interactions of MYC in the module and filtered the proteins according to available 3-D structure information in PDB (Berman et al., 2002), and drug-gene interaction information in geneXpharma (Turanli et al., 2017b). The structures of 60 proteins out of 116 proteins were found in PDB, and drug interactions were identified for 39 proteins with available structural information. Durg repositioning simulation through geneXpharma resulted in 651 drugs associated with 39 target proteins. Then, these drugs were filtered according to FDA-approval, association with CRC in literature, and having the 3-D structures on PubChem (Kim et al., 2019). Finally, we identified eight drugs (abacavir, voriconazole, exemestane, nortriptyline hydrochloride, tolcapone, bromocriptine mesylate, ribociclib, and theophylline) targeting 4 proteins (PML, GSK3B, CDKN2A, HDAC2) (Figure 4).

### *In silico* Investigation of Potential Drug-Target Interactions by Molecular Docking Simulations

**FIGURE 5:**
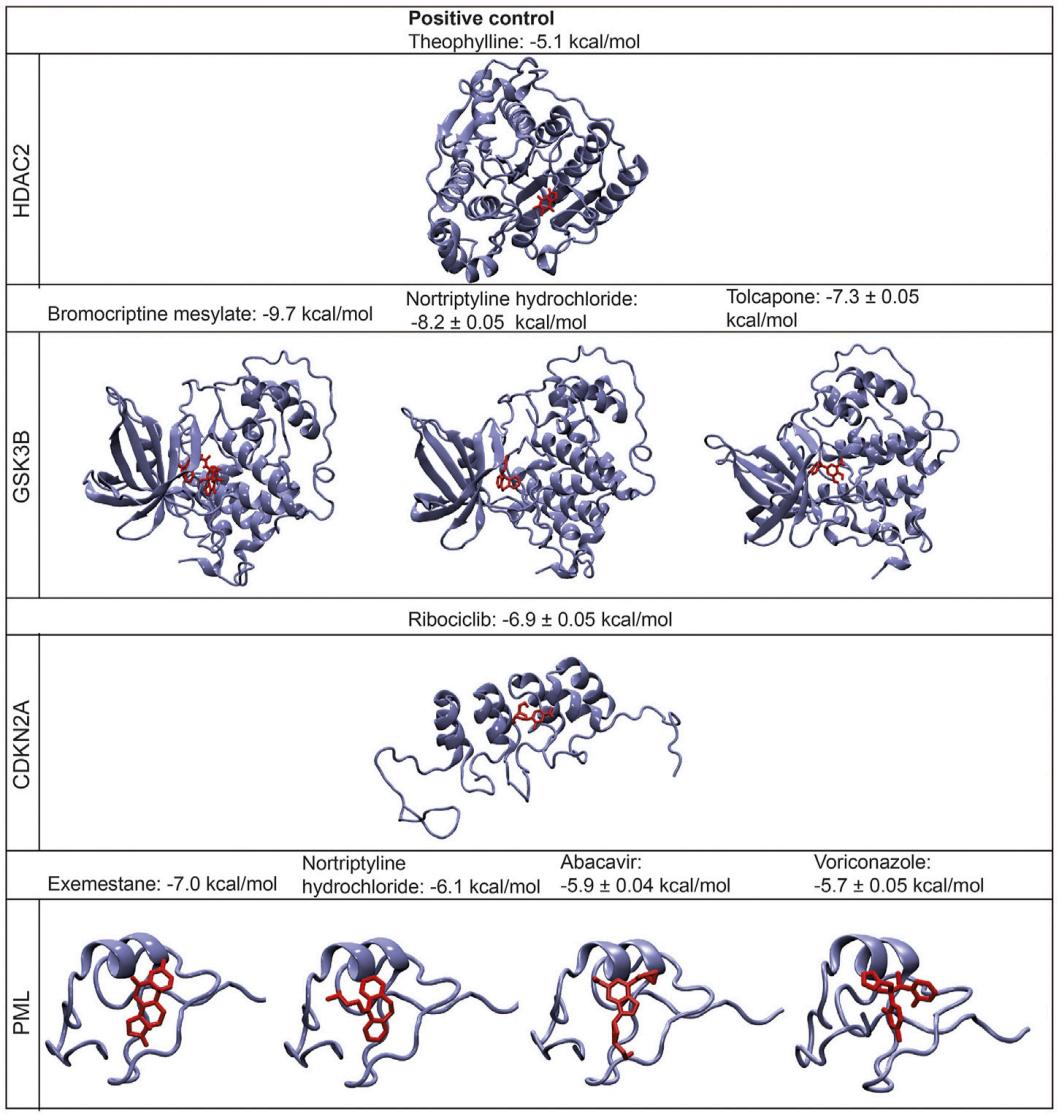
The structures of the bound protein-ligand complexes for each drug target and their binding affinities (mean ± standard deviation (S.D).

*In silico* validation studies were carried out to evaluate the potential candidate drugs for the therapeutic strategies in CRC prior to *in vitro* viability assays. For this purpose, the 3-D structures of target proteins, i.e., 1DC2 (CDKN2A), 6Y9R (GSK3B), 6WBZ (HDAC2), and 5YUF (PML), and candidate drugs were retrieved from PDB (Berman et al., 2002) and PubChem (Kim et al., 2019), respectively, and the binding affinities of the drugs to their targets were estimated via protein-ligand molecular docking simulations using Autodock Vina (Trott and Olson, 2019) (Figure 5).

The binding affinity of the positive control case (theophylline) was predicted to be −5.1 kcal/mol. The most significant binding affinities were obtained for bromocriptine mesylate, nortriptyline hydrochloride, tolcapone, and exemestane, which were predicted as −9.7, −8.2, −7.3, and −7 kcal/mol, respectively. Similarly, the binding affinities of ribociclib, abacavir, and voriconazole were −6.9, −5.9, −5.7 kcal/mol, respectively. Besides, the binding affinity of nortriptyline hydrochloride which targets PML protein was -6.1 kcal/mol. According to the molecular docking simulations, all potential drugs (bromocriptine mesylate, tolcapone, nortriptyline hydrochloride, ribociclib, exemestane, voriconazole, and abacavir) display significantly higher binding affinities when docked to their target proteins, compared to the positive control case (Figure 5). The details of the top-scoring pose of five docking simulations for each ligand are displayed in Supplementary Figure S1.

### *In vitro* Cell Viability Assay of Repurposed Drugs

To have an insight into the antitumor potential of drugs for CRC treatment, *in vitro* cell viability assay was performed in HCT-116 (carcinoma) cell line. Concentration ranges as 10–200 uM for abacavir and tolcapone, 5–100 uM for ribociclib, 200–500 uM for exemestane, voriconazole, and bromocriptine mesylate, 10–50 µM for nortriptyline hydrochloride, and 20–500 µM for theophylline were tested to find out the IC_50_ values in HCT-116 cells.

**FIGURE 6:**
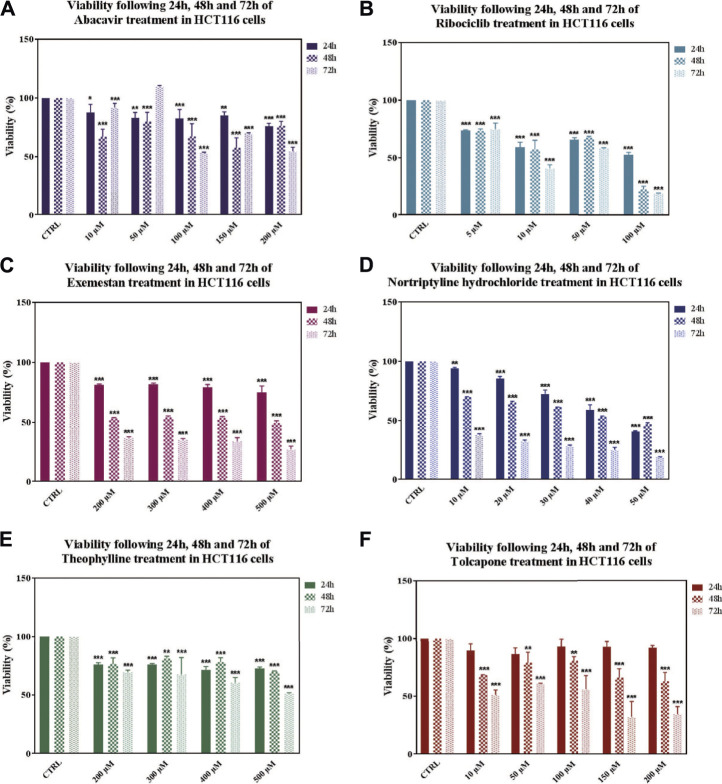
Effects on viability in HCT116 cells following 24, 48 and 72 h of drug treatments. Data denote mean ± S.D. *p < 0.05 vs. CTRL (*n* = 3); **p < 0.01 vs. CTRL (*n* = 3); ***p < 0.001 vs. CTRL (*n* = 3). Viability following of Abacavir **(A)**, Ribociclib **(B)**, Exemestane **(C)**, Nortriptyline **(D)**, Theophylline **(E)**, Tolcapone **(F)** treatment in HCT116 cells.

After 24, 48, and 72 h drug treatments, IC_50_ values were determined as 100 μM at 72 h for abacavir, 100 μM at 72 h for ribociclib, 500 μM at 48 h for exemestane, 40 μM at 48 h for nortriptyline hydrochloride, 500 μM at 72 h for theophylline and 10 μM at 72 h for tolcapone (Figure 6). Bromocriptine mesylate and voriconazole did not decrease viability at tested concentrations and time points.

**FIGURE 7:**
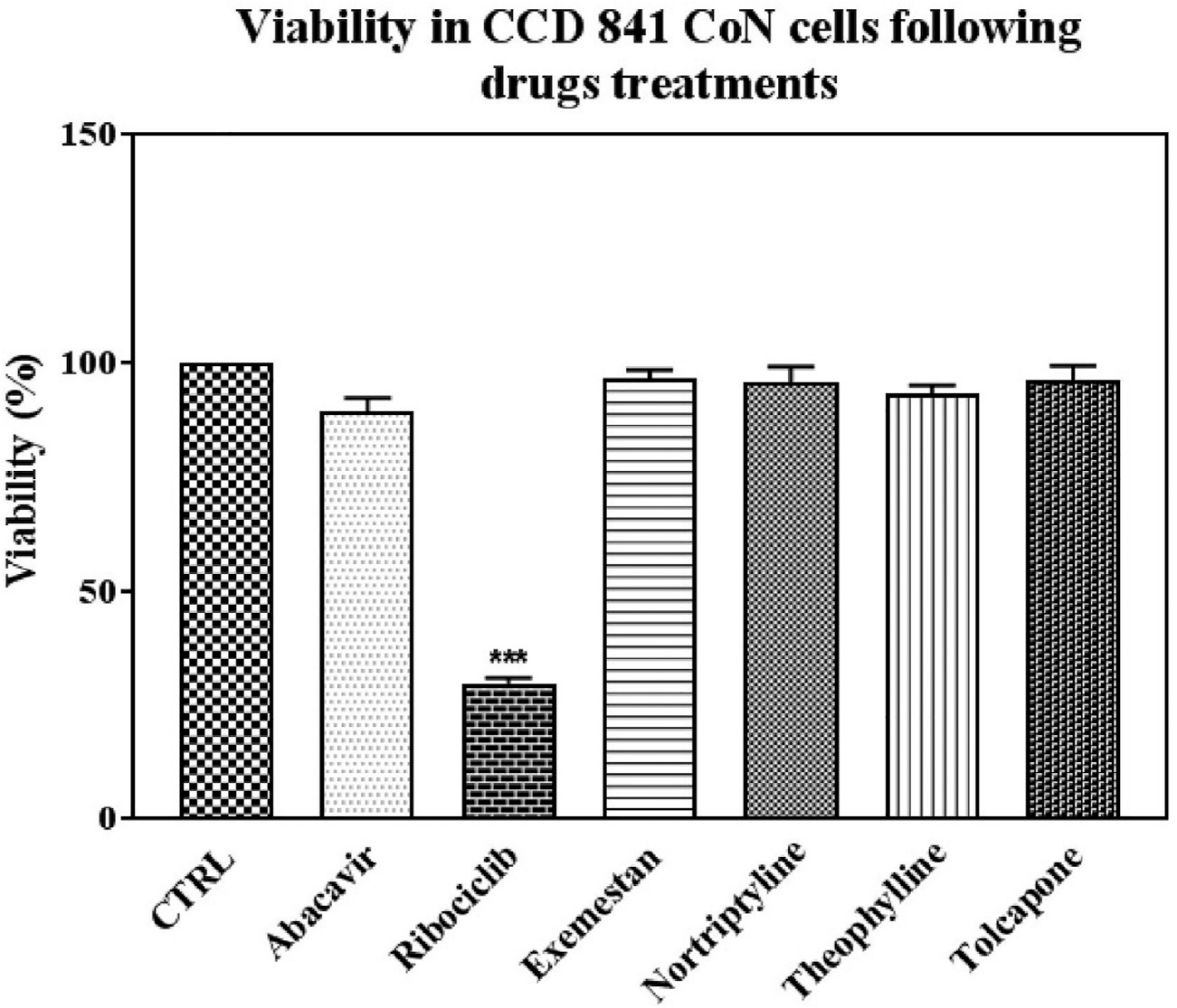
Effects on viability in CCD 841 CoN cells following drug treatments in IC50 conditions. Data denote mean ± S.D. ***p < 0.001 vs. CTRL (*n* = 3). Abacavir, 100 uM and 72 h; Ribociclib, 100 uM and 24 h; Exemestane, 500 uM and 48 h; Nortriptyline, 40 uM and 72 h; Theophylline 500 uM and 72 h; Tolcapone, 10 uM, and 72 h treatment in CCD 841 CON cells.

In addition, the toxicity of the drugs was tested in and CCD-841-Con healthy epithelial cells in the obtained IC_50_ values. Ribociclib was the only drug that caused death in healthy cells when compared to control (Figure 7).

## Discussion

In the present study, we aimed to identify potential systems biomarkers and drug candidates for the treatment of CRC. To this end, we considered the transcriptome profiles of colorectal adenomas and carcinomas together, as colorectal carcinomas predominantly arise from adenomas, and applied the differential interactome approach for the first time in CRC to identify prognostic system biomarkers and drug repositioning candidates. The potential of drug candidates was demonstrated by *in silico* molecular docking simulations and *in vitro* cytotoxicity assays on CRC and healthy cell lines. In summary, 24 modules were found in both GEO and TCGA datasets, six of which showed tumor prognosis. As a result of the detailed evaluation of modules within the relevant literature, MYC, a transcription factor that coordinates many biological processes, came to the fore in all aspects. The MYC-centered module (mMYC) showed high prognostic performance in both datasets; MYC was significantly overexpressed in human cancers (Dang et al., 2009), and it promotes cell proliferation, sensitizes to apoptosis, and induces cells to undergo apoptosis (Massó-Vallés and Soucek, 2020). Therefore, it was promising to target MYC and its interacting partners as therapeutic targets in cancer therapy. On the other hand, previous studies reported that targeting MYC was problematic (Wolf and Eilers, 2020) and direct targeting of MYC for cancer therapy has not been proposed as a rational strategy (Chen et al., 2018); therefore, we hypothesized that breaking the bond between MYC and its interacting partners would be a promising strategy for the treatment of CRC. For this reason, we focused on four MYC-interacting proteins, i.e., Promyelocytic leukemia protein (PML), Glycogen synthase kinase-3 beta (GSK3B), Cyclin-Dependent Kinase Inhibitor 2A (CDKN2A), and Histone deacetylase 2 (HDAC2), which were reported to be mis-regulated in cancer in previous studies (Guan and Kao, 2015; Domoto et al., 2016; Zhao et al., 2016; Shan et al., 2017).

According to the GeneCards database (Stelzer et al., 2016), the PML protein is a member of the triple motif family (TRIM). This phosphoprotein localizes in nuclear bodies where it functions as a transcription factor and tumor suppressor by preventing cells from growing and dividing in an uncontrolled manner. The tumor suppressor PML regulates cell cycle, apoptosis, senescence, migration, angiogenesis, and DNA repair pathways as well as the p53 response to oncogenic signals (Guan and Kao, 2015). GSK3B protein is a serine-threonine kinase that belongs to the glycogen synthase kinase subfamily. It is a regulator of glucose homeostasis and plays tumor promoting roles in cell survival, evasion of apoptosis and proliferation (Domoto et al., 2016). CDKN2A has two different promoters, which are involved in the retinoblastoma protein (Rb) and p53 pathways, and acts as an inhibitor of CDK4/6 kinase. CDKN2A contributes to the regulation of cell cycle progression by inhibiting the S phase. It binds to CDK4/6, inhibiting cyclin D-CDK4/6 complex formation and phosphorylation of Rb family members so preventing exit from G1 phase of the cell cycle (Romagosa et al., 2011). HDAC2 protein plays role in gene transcription, DNA repair, immune stability, and related signaling pathways (Shan et al., 2017). Moreover, the PML protein showed a high expression pattern in endothelial cells and peripheral nerves/ganglia, and similarly, CDKN2A protein showed a high expression pattern in glandular cells and peripheral nerve/ganglion. When HDAC2 protein was examined in Human Protein Atlas, it showed a high expression pattern in glandular cells and an intermediate expression pattern in endothelial cells and peripheral nerve/ganglion. GSK3B protein also showed an intermediate expression pattern in glandular cells and peripheral nerve/ganglion and was detected in plasma (Uhlen et al., 2015).

Among the proposed drugs, abacavir, voriconazole, exemestane, and nortriptyline hydrochloride targeted the PML protein; whereas tolcapone, bromocriptine mesylate, and nortriptyline hydrochloride targeted GSK3B. CDKN2A was the target protein for ribociclib. Additionally, theophylline targeted the HDAC2 protein. Recently, researchers investigated the antitumor effects of the reverse transcription inhibitor abacavir in several studies. Carlini and coworkers (Carlini et al., 2010) investigated the effect of abacavir in prostate cancer cell lines and observed that abacavir reduced cell growth, migration, and invasion processes. Another study investigated the effect of abacavir and its combination with other reverse transcriptase inhibitors in breast cancer cells and found an increase in apoptosis and a decrease in migration in treated cells (Sekeroglu et al., 2021). On the other hand, Exemestane is already used in breast cancer, but Koutras and colleagues (Koutras et al., 2009) observed the antiplatelet effect of Exemestane also in lung cancer cells. Moreover, nortriptyline, a tricyclic antidepressant used to treat depression, however, has antineoplastic activity in various cancers such as bladder, prostate, myeloma (Pan et al., 2010; Mao et al., 2011; Yuan et al., 2015). In addition, recent studies have shown that tolcapone, a catechol-O-methyltransferase inhibitor used in Parkinson’s disease, decreases cell viability in lung cancer and neuroblastoma cell lines (Forester and Lambert, 2014; Maser et al., 2017).

According to DrugBank (Wishart et al., 2006), abacavir was a reverse transcriptase inhibitor and was used against Human Immunodeficiency Virus Type 1 (HIV-1). It targets HIV reverse transcriptase by forming the pharmacologically active compound carbovir 5′-triphosphate, which is an analogue of guanosine. Voriconazole is used to treat severe fungal infections. It binds to 14-alpha-sterol demethylase, which is known as CYP51, and inhibits the demethylation of lanosterol as part of the ergosterol synthesis pathway in yeast and other fungi. Exemestane is an irreversible steroidal aromatase inactivator structurally related to the natural substrate androstenedione that has been used to treat estrogen receptor-positive breast cancer. It acts as a false substrate for the aromatase enzyme and is processed into an intermediate that irreversibly binds to the active site of the enzyme and causes its inactivation. Nortriptyline hydrochloride is a tricyclic antidepressant and is used to treat depression by inhibiting serotonin and norepinephrine reuptake in neuronal cell membranes. It also exerts antimuscarinic effects through its action on the acetylcholine receptor. Tolcapone is a selective and reversible inhibitor of catechol-O-methyltransferase (COMT). It is used for the symptomatic treatment of Parkinson’s Disease besides levodopa/carbidopa therapy. Bromocriptine mesylate is a dopamine D2 receptor that stimulates centrally located dopaminergic receptors, resulting in a number of pharmacological effects. It is used for signs and symptoms of Parkinsonian syndrome. Ribociclib is a cyclin-dependent kinase inhibitor and helps slow the growth of cancer cells by inhibiting CDK4/6 by arresting cells at the G1 checkpoint, preventing tumor cells from proliferating. Theophylline is used to treat lung diseases such as asthma. It has two distinct effects in the airways of patients with reversible obstruction; smooth muscle relaxation and suppression of airway response to stimuli. Besides, it was investigated whether these drugs had been used previously in CRC. With the exception of theophylline, the drugs were novel in CRC. Theophylline was used as a positive control in this study.

Drugs that are candidates for the targets (PML, CDKN2A, GSK3B, and HDAC2) were further evaluated using the current literature and screened for association with CRC. Theophylline was used in SW480 cell lines and the IC_50_ value of theophylline was found to be 10^−4^ M at 48 h in rectal cancer (Peng et al., 2018). In our study, theophylline showed its IC_50_ effect at 500 µM concentration at 72 h after viability assay in the carcinoma cell line. The difference in cell lines might have caused the difference in inhibitory concentrations. It was also observed that ribociclib, a cyclin-dependent kinase inhibitor that slows cancer cell growth by inhibiting CDK4/6 proteins, significantly decreased viability in the healthy cell line in addition to its effect in the carcinoma cell line. We speculate that the possible reason for the selectivity between healthy and CRC cells may be that CRC cells are less dependent on the CDK4/6 axis for proliferation and survival than healthy cells.

## Conclusion

This study aimed to find new therapeutic targets and drugs for the treatment of CRC. There were 24 common protein modules, whose Kaplan-Meier plots showed that six modules were prognostically important. In addition, functional enrichment analysis of the modules revealed that they were involved in signaling pathways related to cancer and metabolism. Molecular docking results showed that each potential drug and respective protein has significantly higher binding affinities compared to the positive control. Four of the drug candidates (abacavir, exemestane, nortriptyline hydrochloride, tolcapone) showed statistically significant inhibition profiles on the CRC cell line. The efficacy of four of these drugs on CRC cell lines was demonstrated in this study for the first time in the literature. Therefore, they can be used for further studies. The positive control (theophylline) showed that our methods can be used as new therapeutics in cancer therapy. Our results may provide new and complementary strategies for the treatment of CRC. On the other hand, these results should be supported by further experiments to elucidate the mechanisms of drug action in colorectal cancer cells. Moreover, our results highlight the value of studying DIPs to propose potential therapeutics.

## Data Availability

Publicly available datasets were analyzed in this study. This data can be found here: GSE8671-https://www.ncbi.nlm.nih.gov/geo/query/acc.cgi?acc=GSE8671; TCGA-COAD- https://portal.gdc.cancer.gov/projects/TCGA-COAD; TCGA-READ- https://portal.gdc.cancer.gov/projects/TCGA-READ.
