# Supplementary figures and images for: Differential Interactome Based Drug Repositioning Unraveled Abacavir, Exemestane, Nortriptyline Hydrochloride, and Tolcapone as Potential Therapeutics for Colorectal Cancers
Source: Front Bioinform. 2021 Sep 14;1:710591. doi: 10.3389/fbinf.2021.710591 (PMC9581026; doi:10.3389/fbinf.2021.710591)

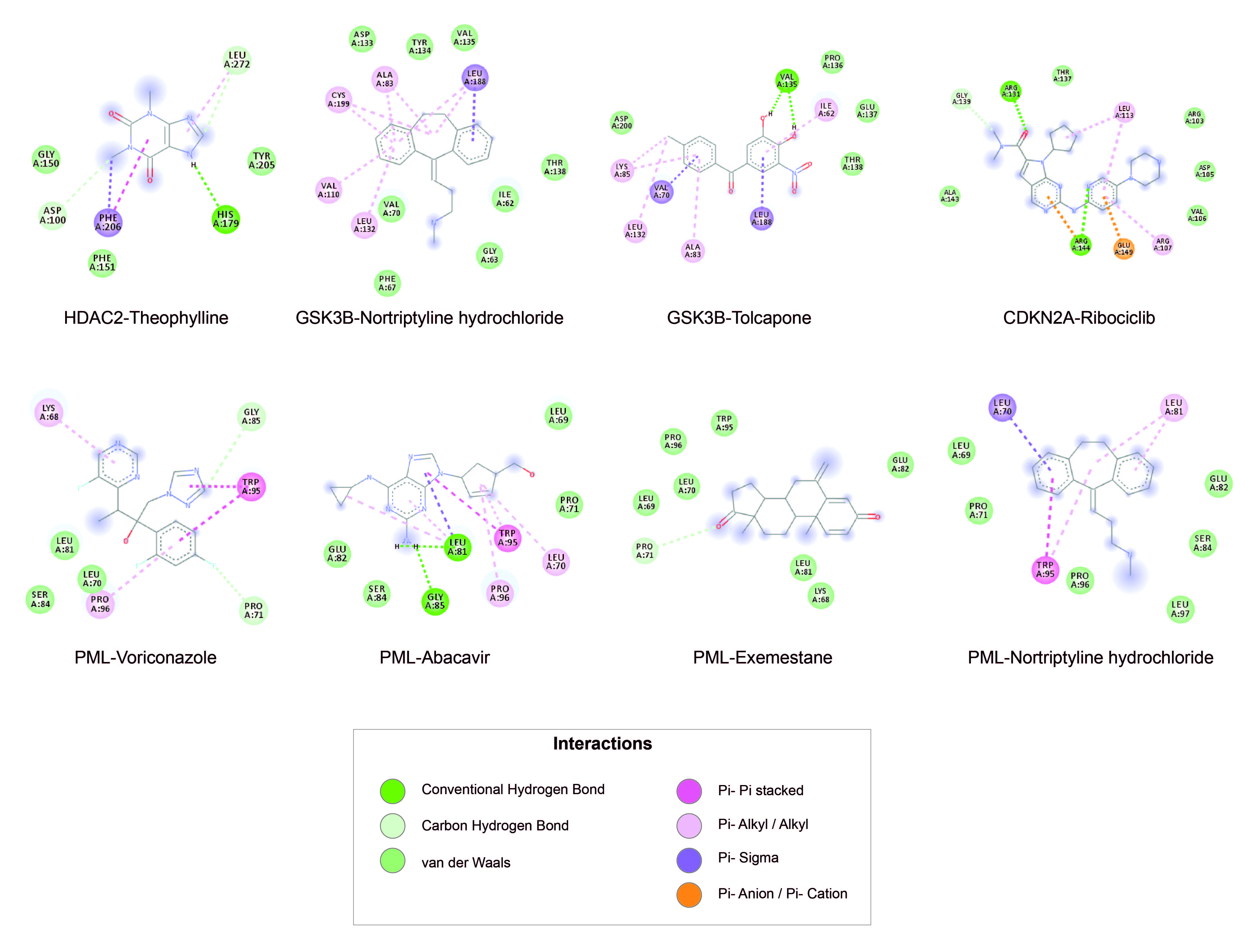

Supplement: Supplementary file 1 [file Image1.JPEG]
